# 

**Published:** 1915-04

**Authors:** 


**Notice to Subscribers in Arrears.** We cannot put it all on the advertisers.

